# Statistical Behavior of the Rank-order and Tiering Metric of the 2020 Centers for Medicare and Medicaid Services Organ Procurement Organization Rule

**DOI:** 10.1097/TXD.0000000000001974

**Published:** 2026-07-07

**Authors:** J. Thomas Rosenthal

**Affiliations:** 1 Department of Urology, UCLA Medical School: University of California Los Angeles David Geffen School of Medicine, Los Angeles, CA.

## Abstract

**Background.:**

The 2020 Centers for Medicare and Medicaid Services (CMS) Organ Procurement Organization (OPO) rule used an estimate of OPO organ donor potential that included contraindications to organ donation, termed cause, age, and location consistent with donation (CALC). CMS asserted that even though CALC was inaccurate, it could still be used to compare OPOs because it highly correlated with CALC-adjusted to exclude the contraindications. Quantitative bias analysis is required to determine whether CALC can, in fact, be reliably used to make certification decisions under the rule.

**Methods.:**

A Monte Carlo simulation was carried out with 10 000 simulations using statistical constraints defining the relationship between CALC and CALC-adjusted applied to the published data in the rule.

**Results.:**

In every simulation, at least 2 OPOs changed tier assignment, with a median of 10 OPOs changing tiers across the 10 000 simulations. Several validations were carried out to confirm the results. All the tier changes were in OPOs that were closely adjacent to one of the cutoffs that determined tier assignment.

**Conclusions.:**

CALC, as applied in the 2020 OPO rule, possesses a level of stochastic instability that exceeds the threshold for accurate regulatory decisions. Its use will result in misclassification of a significant number of OPOs.

In the 2020 Centers for Medicare and Medicaid Services (CMS) Organ Procurement Organization (OPO) rule, cause, age, and location consistent with donation (CALC), determined from causes of death on death certificates obtained from the Centers for Disease Control and Prevention Multiple Cause of Death—All Counties database, was used to determine each OPO donation service area’s potential donor number used to calculate donation and transplantation rates.^1^ Public comments observed that CALC includes cancers, infections, and nonventilated cases that are contraindications to organ donation, that these contraindications are not proportional across OPOs, and that this differential selection bias would cause inaccurate OPO performance assessments. A single article was referenced (hereafter called the reference article), which asserted that because CALC and CALC-adjusted for contraindications were highly correlated, the assignment of OPOs to 1 of 3 tiers that determines certification would be identical regardless of which measure was used.^2^ In other words, the degree of differential selection bias would not lead to systematic biases in performance assessments.

Tier assignments are highly consequential: assignment to tier 3 results in automatic OPO decertification by CMS, which means the OPO ceases to exist; tier 1 assignment means automatic recertification; and tier 2 assignment allows other OPOs to compete for the service area. For example, if an OPO was tier 2 under CALC but switched to tier 1 under CALC-adjusted, it means that the metric used in the rule would have resulted in a high-functioning OPO not being certified that should have been certified.

A subsequent study confirmed that the 2 methods are highly correlated but, unlike the reference article, it rank-ordered OPOs using both measures and showed that 20%–29% of OPOs changed tier assignments when CALC-adjusted was used instead of CALC.^3^ Despite these results, the rule has not been modified and will still be used in 2026 to make OPO certification and decertification decisions. The question of whether CALC and CALC-adjusted produce equivalent results has important public policy implications and important implications for how organ donation functions in the United States. It therefore warrants a definitive answer.

It is virtually impossible to obtain state hospital discharge data for every OPO, so the determination of tier assignment accuracy cannot be tested directly with data from all OPOs. Because the statistical relationship between the donor potentials determined by CALC and by CALC-adjusted was well defined in both articles, a Monte Carlo simulation can be applied to all 58 OPOs to assess whether the high degree of statistical correlation observed in both studies actually results in equivalence of the 2 methods.^4^ Quantitative bias analysis was described as “essential when a report makes action or policy recommendations and the decisions are sensitive to biases.”^5^ This is clearly the case for the OPO rule.

## MATERIALS AND METHODS

The reference article determined the relationship between CALC and CALC-adjusted using hospital-coded discharge data obtained from the Agency for Healthcare Research and Quality on 48 OPOs. It showed a Pearson coefficient of 0.97, Bland-Altman analysis showing 97.9% of paired values were within 1.96 SDs of the mean difference, a Spearman correlation coefficient of 0.95, and a Lin’s concordance correlation coefficient of 0.97 based on standardized SDs. Individual OPO data were presented graphically. To determine the correlation equation, values were extracted from the graph, entered into a table, and the correlation equation for the relationship between CALC and CALC-adjusted was determined: Donation rate-CALC-adjusted = 1.9449 × Donation rate–CALC minus 0.007374.

Rank-order and tier assignments of 58 OPOs were taken from the 2020 CMS OPO rule, which used estimates of donor potential, CALC, obtained from the 2018 CMS Multiple Cause of Death Files, rank-ordered OPOs from highest to lowest by 95% 1-sided upper confidence interval calculated by the Wilson score interval with continuity correction, and assigned each OPO to 1 of 3 tiers.^1^ The highest tier OPOs have 1-sided 95% confidence intervals greater than the prior year’s top 25th%tile. The lowest-tier OPOs have 1-sided 95% confidence intervals less than the prior year’s median. The middle tier are those less than the top 25th%tile but above the median. The cutoffs for 2018 were 11.37% (tier 1–2) and 9.72% (tier 2–3).

CALC-adjusted was then calculated for all 58 OPOs by generating synthetic scores using a Monte Carlo procedure that adds Gaussian-like noise to the regression equation, epsilon, orthogonalized to preserve the regression structure and conforming to the statistical constraint of a Pearson correlation *r* = 0.97, Bland-Altman showed that 97.9% of paired values were within 1.96 SDs of the mean difference, with a Spearman correlation coefficient of 0.95, and Lin’s concordance correlation coefficient of 0.97 based on standardized SDs. The 95% 1-sided confidence intervals were calculated using the Wilson score interval with continuity correction, and OPOs were rank-ordered and assigned tiers using cutoffs calculated using the regression equation: 21.20% (tier 1–2) and 18.03% (tier 2–3). OPOs with different tier assignments, when donation rates were determined by CALC-adjusted versus when measured by CALC, were identified for each simulation.

The process was repeated with 10 000 independent simulations to determine the CALC-adjusted donation rate, conforming to the correlation equation and associated statistical constraints using Excel. The mean number of tier changes per 10 000 simulations, the distribution of tier-change counts, the number and probability of simulations with zero tier changes, and entity-specific tier-change rates, defined as the proportion of simulations in which a given entity changed classification, and the probability of zero tier changes were calculated.

Because CALC and CALC-adjusted were extracted from a graph, a sensitivity analysis was carried out to identify the range of possible correlation equations that would be possible, consistent with the following constraints: each observed value was allowed to vary by ±2%; the relative rank-order of the entities was preserved; and the resulting paired observations satisfied a Pearson correlation coefficient of 0.97.

Under these constraints, the feasible regression equations relating CALC to CALC-adjusted from article 1 were bounded by 2 extreme solutions corresponding to the minimum and maximum allowable regression slopes. The minimum and maximum correlation equations for donation rates were Donation rate-CALC-adjusted = 1.9362 × Donation rate-CALC minus 0.0065 and Donation rate-CALC-adjusted = 1.961 × Donation rate-CALC minus 0.0090. Ten thousand simulations were each run for both maximum and minimum correlations.

A simulation with 10 000 independent simulations was also run using the correlation equation from article 2, which was determined from precise values of CALC and CALC-adjusted based on data obtained on 28 OPOs from Agency for Healthcare Research and Quality: Donation rate-CALC-adjusted = 4.5851 × Donation rate-CALC minus 0.03653 (regression equation provided by the author).^3^

To be certain that the 2018 rank-order was not an anomaly, the simulation was run on rank-order and tier assignments for the 55 remaining OPOs from 2023 data^6^ with cutoffs defined by the 2022 25%tile and median, using the correlation equation and statistical constraints from the reference article.

Each OPO’s distance from the nearest cutoff was calculated by dividing the difference of its 95% confidence interval and the nearest cutoff (either 9.72% or 11.37%) by the nearest cutoff. This percentage difference was plotted against the tier-change frequency from the simulation, which used the reference equation and its statistical constraints.

The simulation procedure was validated by manual calculation in spreadsheets of 3 simulation iterations, 1 each of high, medium, and low numbers of tier changes, which confirmed the calculation of CALC-adjusted from the correlation equations; the calculation of the 95% 1-sided confidence interval using the Wilson formula; the identity and number of OPOs with tier changes; and verified the Pearson constraint for sampled runs. Additional validation was obtained by replication of the 10 000 simulations under different random seeds to confirm stability of estimated flip rates and zero-flip probabilities. Finally, additional validation was obtained under non-Gaussian error assumptions using heavy-tailed Student *t*-distributed errors (*df* = 5) and the same statistical constraints.

ChatGPT (OpenAI) was used as a computational aid to assist in implementing Monte Carlo simulation code. The study design, statistical assumptions, parameter selection, simulations, and validation of simulation results were performed independently by the author, who takes full responsibility for the accuracy of the analysis. No language used in the article was generated by artificial intelligence.

This study included no animal or human participants and did not require approval from an ethics board.

## RESULTS

The results are presented in Table 1, showing the mean, median, minimum, maximum, and percentage of tier changes for each analysis. Each of the 10 000 simulations produced 0 (zero) instances where no OPO changed tier assignment. For the reference correlation equation, the median number of tier changes was 11 (19% of 58 OPOs). The upper bound on probability was estimated using the rule of 3 (*P* < 3/N),^7^ with N = 10 000, *P* < 0.0003, so the probability that no OPOs change tiers is <0.03%, corresponding to the odds of zero tier change being greater than approximately 1 in 3000.

**TABLE 1.** Tier changes for each scenario with 10 000 simulations showing mean, median, minimum, and maximum tier changes and percentage of simulations with tier changes

Scenario	Mean	Median	Minimum	Maximum	% Simulations with tier change
Reference equation	10.71	11	2	21	100%
Reference reseeded	10.67	11	2	22	100%
Reference Student *t* test	10.06	10	1	20	100%
Minimum equation	10.74	11	3	20	100%
Maximum equation	10.65	11	2	20	100%
Lopez/Schold equation	12.30	12	4	22	100%
2023 Data	11.39	11	7	15	100%

**FIGURE 1.**
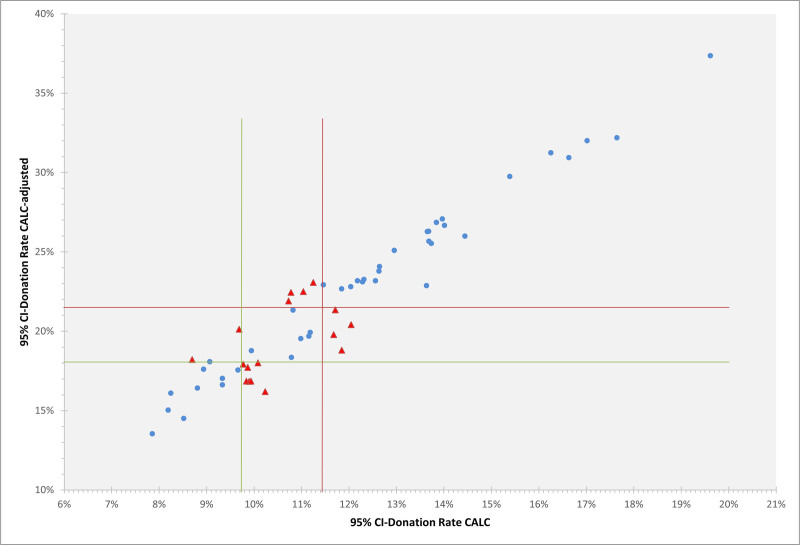
Single simulation comparing donation rates obtained using CALC (x-axis) and CALC-adjusted (y-axis) as denominators. The purple lines are the tier 1–2 cutoffs; the green lines are the tier 2–3 cutoffs. The red triangles are Organ Procurement Organizations (OPOs) that change tier assignments. CALC, cause, age, and location consistent with donation; CI, confidence interval.

Figure 1 shows details from 1 simulation. The values for CALC are those from Table 1 in the 2020 Rule. The simulation conforms to correlation equation 1, and the associated statistical constraints. In this simulation, 17 of 58 OPOs change tier assignments. All the changes shown by red triangles occur very close to the cutoff edges, where the donor percentages are tightly clustered and very small changes in donor potential are sufficient to move an OPO up or down in rank-order to change tiers.

**FIGURE 2.**
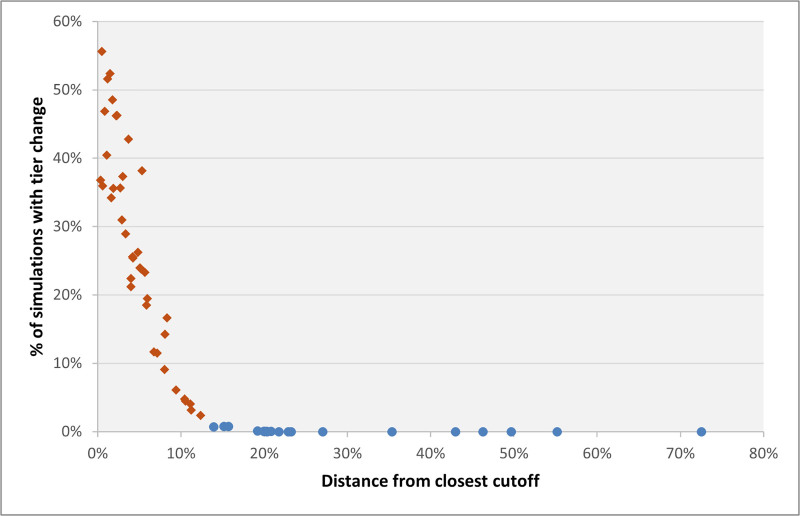
Tier changes for 58 Organ Procurement Organizations (OPOs) as a function of distance from the closest cutoff. The 19 with <1% of simulations resulting in tier changes are shown in blue circles. The 39 with >1% of simulations resulting in tier changes are shown in purple diamonds.

Figure 2 shows the frequency distribution of tier changes by OPO distance from the nearest cutoff for the 58 OPOs. There were 16 OPOs at the top of the rank-order and 3 OPOs at the bottom of the rank-order that changed tiers in <1% of simulations. The other 39 have increasing probability of changing tiers the closer they are to one of the cutoffs, with 22 OPOs that were within 5% of a tier cutoff having 20%–56% chance of tier change. The correlation is less than perfect, because donation rate for CALC-adjusted is determined by the donation rate for CALC, while the rank-order and tier assignments are determined by the 1-sided 95th%tiles, which are sensitive to size.

Manual calculation of 3 simulation iterations in spreadsheet form confirmed the accuracy of the calculation of CALC-adjusted from the correlation equations, the accuracy of the calculation of the 95% 1-sided confidence interval using the Wilson formula, confirmed the identity and number of OPOs with tier changes, and verified that the Pearson and Spearman correlations and the Bland-Altman and Lin constraints were met. Three simulations under different random seeds and the simulation using Student *t* test produced zero-flip probabilities and nearly identical tier-change rates.

## DISCUSSION

A critical question in the 2020 OPO rule was whether CALC, the measure of OPO donor potential as the denominator in determining donation rates, is accurate enough to make certification decisions. CMS acknowledged that CALC is inherently inaccurate as a measure of real donor potential but asserted that it could still be used to compare relative performance.^8^ In other words, CALC does not have to accurately measure OPO donor potential, but it does have to perfectly measure tier assignments of all 58 OPOs. CMS concluded that the near-perfect correlation of CALC and CALC-adjusted would produce identical tier assignments, thereby justifying CALC’s use in the rule.

Monte Carlo analysis shows that near-perfect correlation does not produce identical tier assignments to CALC-adjusted. Across 10 000 independent simulations, 11 OPOs changed tiers on average and every simulation produced at least 2 OPOs whose tier assignment changed when CALC-adjusted was used compared with CALC. Based on standard statistical inference, this establishes odds of at least several thousand to one against the possibility that no tiers would change. In practical terms, tier changes are not merely possible; they are virtually inevitable.

The reason that near-perfect correlation does not produce identical tier assignments is a function of the structure of the data and the placement of the cutoffs: 38% of OPOs lie within 5% of one of the tier cutoffs with 4 lying within 1% of a tier cutoff, well within the statistical correlation constraints and highly susceptible to tier change. The rank-order tiering system is highly sensitive to trivial variations in the donor potential denominator. As an example, the published rule shows Texas Organ Sharing Alliance, San Antonio Texas, having a potential donor number of 1913 and is designated as tier 2. A difference in donor potential of <0.7%, well within the statistical constraints of allowable epsilons in the simulations, would change its tier assignment, which explains why it changes tiers in the analysis in almost 60% of simulations.

The stability of the top and bottom and the fragility of the middle is a common feature of closed rank-order systems.^9^ This might suggest that CALC could be used if a more standard cutoff was used, like SD below the mean. However, CALC-adjusted is, itself, not a true measure of donor potential, and the data here cannot assess the probability that the 3 stable OPOs at the bottom of this rank-order analysis will or will not rise above a SD below the mean cutoff if donor potentials were directly measured and risk-adjusted.

Using CALC to determine OPO donor potential is only 1 problem undermining the credibility of the CMS rule metric. Other studies have recently been published that highlight different aspects of the rule metric to accurately differentiate OPO performance, including bias against large OPOs,^10^ failure to risk-adjust for factors known to impact performance,^11^ and instability in tier assignments from 1 y to the next, described as fragility.^12^

Several questions should be addressed. The accuracy of the correlation equation of the reference article could be questioned, as it was derived from a graph. However, the range of correlation equations possible under the statistical constraints produces the same probability of tier changes. Concerns that OPO rank-orders could have materially changed since 2018 are addressed by a simulation using the latest CMS published OPO data. The reason that the correlation equations are significantly different between the 2 articles is that both CALC and CALC-adjusted are dependent on how many coded diagnoses are used. CALC from hospital discharge data using all coded discharge diagnoses is approximately 4 times CALC that only uses the primary discharge diagnosis.^13^ It is not stated in the reference article how many discharge codes were used, but it is likely different than the 10 used by Lopez-Schold. Another concern could be that the reference article used state level detail for California and Florida, each with 4 OPOs. However, if county-level detail had been used, additional variability would have been introduced, lowering the degree of correlation and increasing, rather than decreasing, the probability of tier changes.

A Monte Carlo simulation that compared CALC and CALC-adjusted using well-defined statistical constraints applied to the CMS OPO rule demonstrates that there is sufficient differential selection bias to cause systematic biases in performance assessments. CALC, as applied in the rule, possesses a level of stochastic instability that exceeds the threshold for high-stakes regulatory decision-making. Its use will result in the incorrect categorization of a large number of OPOs with a high degree of certainty, making the development of alternative metrics of OPO performance imperative.
